# Systematic review of neighborhood socioeconomic indices studied across the cancer control continuum

**DOI:** 10.1002/cam4.4601

**Published:** 2022-02-14

**Authors:** Kristen A. Sorice, Carolyn Y. Fang, Daniel Wiese, Angel Ortiz, Yuku Chen, Kevin A. Henry, Shannon M. Lynch

**Affiliations:** ^1^ Cancer Prevention and Control Fox Chase Cancer Center Philadelphia PA USA; ^2^ Geography and Urban Studies Temple University Philadelphia PA USA

**Keywords:** cancer incidence, cancer mortality, cancer survival, health disparities, neighborhood deprivation index, socioeconomic status, systematic review

## Abstract

**Background:**

There is extensive interest in understanding how neighborhood socioeconomic status (nSES) may affect cancer incidence or survival. However, variability regarding items included and approaches used to form a composite nSES index presents challenges in summarizing overall associations with cancer. Given recent calls for standardized measures of neighborhood sociodemographic effects in cancer disparity research, the objective of this systematic review was to identify and compare existing nSES indices studied across the cancer continuum (incidence, screening, diagnosis, treatment, survival/mortality) and summarize associations by race/ethnicity and cancer site to inform future cancer disparity studies.

**Methods:**

Using PRISMA guidelines, peer‐reviewed articles published between 2010 and 2019 containing keywords related to nSES and cancer were identified in PubMed.

**Results:**

Twenty‐four nSES indices were identified from 75 studies. In general, findings indicated a significant association between nSES and cancer outcomes (*n* = 64/75 studies; 85.33%), with 42/64 (65.63%) adjusting for highly‐correlated individual SES factors (e.g., education). However, the direction of association differed by cancer site, race/ethnicity, and nSES index.

**Conclusions:**

This review highlights several methodologic and conceptual issues surrounding nSES measurement and potential associations with cancer disparities. Recommendations pertaining to the selection of nSES measures are provided, which may help inform disparity‐related disease processes and improve the identification of vulnerable populations in need of intervention.

## Graphical abstract

After a comprehensive systematic review, 24 unique nSES indices were identified with significant associations only observed between nSES, cancer incidence among non‐Hispanic Whites, and cancer survival for select sites. Findings highlight the complex association between nSES and cancer control outcomes and a need for standardized approaches to nSES measurement and variable selection in future studies.

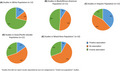

## 1 INTRODUCTION

In the United States, approximately 40% of men and 38% of women will develop cancer in their lifetime.^1^ Abundant research has focused on identifying biological and individual‐level exposures and risk factors for cancer; but recently, there is increasing emphasis on understanding how neighborhood‐level factors, notably neighborhood socio‐economic environment, impact cancer incidence, and mortality.^2^ Neighborhood socioeconomic status (nSES), often defined in terms of the economic (e.g., employment, income), physical (e.g., housing/transportation), and social (e.g., poverty, education) characteristics of a place where a person lives,^3^, ^4^ has been associated with risk for chronic diseases including stroke,^5^ coronary heart disease,^6^ and select cancer outcomes.^7^ Some multilevel conceptual frameworks have been developed to illustrate the various pathways by which nSES can impact cancer outcomes.^2^, ^8^, ^9^, ^10^ For instance, low SES neighborhoods often lack adequate health care resources, which can influence behavioral pathways associated with cancer outcomes, including timely receipt of cancer screening or access to quality care related to risk‐reducing interventions (e.g., smoking cessation).^2^, ^8^, ^9^, ^11^, ^12^, ^13^, ^14^, ^15^, ^16^ In the context of these frameworks, nSES has also been shown to impact biologic pathways implicated in cancer under a chronic stress hypothesis. Specifically, studies show that residents from disadvantaged neighborhoods experience greater emotional stress and constant “wear and tear” on the body that can affect cancer initiation and progression^17^, ^18^ and biologic markers associated with cancer, such as telomere length.^19^ Furthermore, these frameworks consider nSES to be an important contributor to race/ethnic disparities often noted in cancer outcomes, given patients of color often disproportionately live in low resource, disadvantaged areas compared to non‐Hispanic White patients (NHW).^2^, ^8^, ^9^, ^20^, ^21^ Thus, empirical evidence indicates that nSES is important to assess in order to fully understand and ultimately better address cancer health disparities. To evaluate the impact of nSES in relation to various cancer outcomes, researchers often create indices to both define and characterize overall nSES in a single measure. These indices are composite measures that provide a summary score of a neighborhood’s overall employment, education, income, housing, etc.^22^ However, numerous nSES indices exist, and the approaches used to operationalize nSES frequently differ across studies. Although nSES indices often comprise similar domains (e.g., income, employment, education, housing) and utilize similar geographic boundaries (i.e., census tracts), the variables used to represent domains differ enough so that one neighborhood may be considered highly deprived by one index but not another.^10^ Thus, using different nSES indices complicates the ability to draw meaningful conclusions about nSES as a common risk factor for cancer. Given the lack of consensus regarding appropriate measures of disparity, several national and federal organizations have called for a standardized approach to measuring neighborhood and sociodemographic effects.^23^

This review aimed to summarize existing nSES indices in the literature and characterize their associations with outcomes across the cancer continuum (incidence, diagnosis, treatment, mortality) overall, and where possible, by cancer site. In light of studies reporting interactions of race/ethnicity with socioeconomic status on cancer outcomes,^24^ associations between nSES indices and cancer outcomes were also examined by race/ethnicity and individual‐level SES to determine if associations vary by race or index used, and to further identify gaps in the literature. Findings from this review will help clarify the potential role of nSES in contributing to cancer outcomes and inform nSES variable selection in future studies.

## 2 METHODS

**FIGURE 1:**
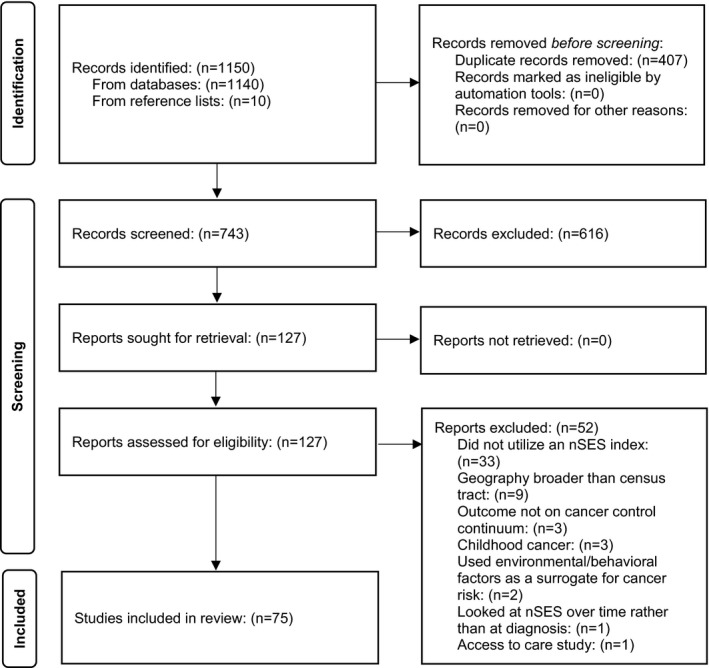
PRISMA flowchart

We conducted a literature review of peer‐reviewed studies to identify nSES measures that were studied in relation to various cancer outcomes. We used the National Library of Medicine’s PubMed search engine, searching articles published from 2010 to 2019. Boolean operator “AND” was used to identify combinations of search terms including neighborhood, neighborhood environment, social environment, contextual, neighborhood deprivation, neighborhood socioeconomic status, neighborhood SES, area‐based SES, macro environment, and segregation (first terms) combined with terms from the cancer control continuum, cancer + risk, incidence, screening, diagnosis, stage, treatment, survival, and mortality (second terms). A manual search of reference lists from reviews and related articles supplemented the electronic search. We followed Preferred Reporting Items for Systematic Reviews and Meta‐Analyses (PRISMA) guidelines for this review (Figure 1). Duplicate manuscripts were deleted. Articles were excluded if they: (1) Did not include a cancer outcome (e.g., studied cancer risk behaviors like smoking); (2) were reviews or theory‐based papers; (3) used nSES measures as a surrogate for individual‐level SES (e.g., used the median household income to represent SES when individual income data were not available); (4) investigated built environment, pollution, or environmental contaminants, not nSES; (5) focused on access to care or supportive care; (6) reported all‐cause mortality, not cancer mortality; (7) were conducted outside the U.S.; (8) investigated broader geographies than census tracts (e.g., county‐level), given prior studies suggest areas larger than a census tract are more susceptible to the modified area unit problem (MAUP) and are likely to result in different associations compared to geographies smaller than a census tract^25^; or (9) did not report relevant statistics (e.g., effect sizes). We identified 1140 articles through the database search and an additional 10 from reference lists. After a preliminary abstract review, 127 full‐text articles were assessed for eligibility; 52 did not meet inclusion criteria, resulting in 75 studies included in this review. Two primary reviewers collected data from the studies, followed by two secondary reviewers for quality assurance; discrepancies were resolved through discussion with the principal investigator and co‐authors.

The studies were categorized based on cancer outcome(s) studied. The overall incidence of each cancer primary was summarized, however, if results were provided for cancer subtypes only (e.g., the incidence of the cardia and non‐cardia gastric cancer rather than overall gastric cancer incidence), the incidence of each subtype was reported.^26^ Only two cancer screening studies were identified^27^, ^28^ across three cancer sites (prostate, colorectal, cervical). Given the small, heterogeneous sample, these results are not shown. Diagnosis studies examined cancer stage, grade, aggressiveness (i.e., in prostate cancer), or hormone receptor status (i.e., in breast cancer). This category was defined, as appropriate for each cancer type, based on the study definition.^3^, ^29^, ^30^, ^31^, ^32^, ^33^, ^34^, ^35^, ^36^, ^37^, ^38^, ^39^, ^40^, ^41^, ^42^ Treatment studies assessed comprehensiveness and time to treatment. Survival studies included an outcome of cancer survival or mortality. nSES indices were defined by either the name given by the original author (e.g., Concentrated Affluence^43^) or the name of the author who first published a study using that index (e.g., Yost Index^44^).

Associations between cancer outcomes and nSES were defined as positive, no association, or inverse using study‐reported effect estimates (i.e., odds ratios, incidence rate ratios, hazards ratios, and *p*‐values (<0.05)). The majority of prior nSES and cancer outcome studies largely rely on reporting statistical significance in terms of *p*‐values, where *p* < 0.05 signify statistical significance. Thus, we used this definition, along with the original authors’ own interpretation or designation of statistical significance, to categorize associations as positive, no association or inverse in this study. We summarized only the results of the final multivariate model presented. Effect sizes are summarized in a publicly available database (Table S1; https://github.com/ksorice/nSES‐Systematic‐Review.) We were unable to conduct a meta‐analysis, given differing coding schemes of nSES across studies (i.e., different indices, quartiles vs. quintiles, etc.).

Indices measuring deprivation and disadvantage were reported inversely so all associations reflected nSES consistently (e.g., low neighborhood deprivation/disadvantage = high nSES). Positive associations between nSES and cancer outcomes include higher nSES being significantly associated with higher cancer incidence, more favorable diagnosis (e.g., lower stage/grade), receiving more comprehensive treatment (e.g., lumpectomy plus radiation vs. lumpectomy alone^45^), and better survival. Because papers often presented multiple sets of results stratified by race, sex, or primary cancer site, and these factors can confound or impact associations between nSES and cancer, studies were additionally assessed for the total number of positive associations, no associations, and inverse associations found overa ll, by cancer site, and by race/ethnic group (White, Black, Asian, Hispanic) summed together to further evaluate nSES associations with cancer outcomes (Tables S2–S6). Most studies are reported in terms of the independent association between nSES and an outcome (i.e., incidence/risk, diagnosis, treatment, survival). If an independent association between nSES and cancer outcome was not available, associations between cancer outcomes and nSES combined with individual SES measures (e.g., nSES/race/education)^46^ or other area‐level measures (e.g., nSES/ethnic enclave)^45^ were assessed. This study was conducted under Protocol #18‐9015 approved by the Institutional Review Board at Fox Chase Cancer Center.

## 3 RESULTS

### 3.1 Overview of nSES Indices

Seventy‐five studies evaluated associations between nSES and cancer control outcomes. A searchable summary of these studies is available online (Table S1). This database allows for comparisons of studies, particularly related to methods and study design that could influence potential associations. Briefly, the majority of studies were cross‐sectional, meaning analysis of nSES was conducted at a single time point (e.g., at the date of diagnosis) (*n* = 70/75 studies). Five studies were longitudinal, comparing cancer outcomes at different nSES periods (e.g., 1998–2002 vs. 2008–2012). The majority of cross‐sectional studies utilized Surveillance, Epidemiology, and End Result Program (SEER) registry data (*n* = 55); 24 studies utilized more detailed individual‐level data (beyond age, race/ethnicity, e.g., smoking history, physical activity, alcohol intake, income). In 37 of 75 studies, the main outcome was cancer survival/mortality; cancer incidence was the outcome in 26 studies. Studies ranged across different US states, but the majority were from California (*n* = 54). Twenty‐four nSES indices were identified (Tables S1 and S2); all were calculated at the census tract level of geography or lower. The Yost Index was most commonly utilized (*n* = 40 studies),^26^, ^31^, ^32^, ^34^, ^35^, ^37^, ^41^, ^45^, ^46^, ^47^, ^48^, ^49^, ^50^, ^51^, ^52^, ^53^, ^54^, ^55^, ^56^, ^57^, ^58^, ^59^, ^60^, ^61^, ^62^, ^63^, ^64^, ^65^, ^66^, ^67^, ^68^, ^69^, ^70^, ^71^, ^72^, ^73^, ^74^, ^75^, ^76^, ^77^ followed by the Concentrated Disadvantage Index (*n* = 6),^33^, ^40^, ^42^, ^78^, ^79^, ^80^ Messer Index (*n* = 4),^3^, ^15^, ^36^, ^81^ and Yang Index (*n* = 4).^82^, ^83^, ^84^, ^85^ Indices were developed using methods including principal components analysis,^3^, ^15^, ^26^, ^30^, ^31^, ^32^, ^33^, ^34^, ^35^, ^36^, ^37^, ^40^, ^41^, ^42^, ^45^, ^46^, ^47^, ^48^, ^49^, ^50^, ^51^, ^52^, ^53^, ^54^, ^55^, ^56^, ^57^, ^58^, ^59^, ^60^, ^61^, ^62^, ^63^, ^64^, ^65^, ^66^, ^67^, ^68^, ^69^, ^70^, ^71^, ^72^, ^73^, ^74^, ^75^, ^76^, ^78^, ^79^, ^80^, ^81^, ^82^, ^83^, ^84^, ^85^, ^86^, ^87^, ^88^, ^89^, ^90^ factor analysis,^27^, ^39^, ^91^, ^92^, ^93^, ^94^, ^95^ principal components analysis plus factor analysis,^96^ *a priori* selection,^29^, ^38^, ^40^, ^78^, ^80^, ^97^, ^98^, ^99^ and weighted quantile sums^28^ that generally characterized indices by eight main domains: income, education, employment, housing, transportation, family structure, demographic data, and other (Table 1). All indices included the income domain, and variables used to represent this domain were relatively consistent (i.e., poverty (n = 18 indices), median household income (n = 11 indices)). Eleven of 24 indices included variables to represent education, employment, and housing, but the variables selected to represent these domains differed.^3^, ^15^, ^26^, ^27^, ^29^, ^31^, ^32^, ^34^, ^35^, ^36^, ^37^, ^39^, ^41^, ^45^, ^46^, ^47^, ^48^, ^49^, ^50^, ^51^, ^52^, ^53^, ^54^, ^55^, ^56^, ^57^, ^58^, ^59^, ^60^, ^61^, ^62^, ^63^, ^64^, ^65^, ^66^, ^67^, ^68^, ^69^, ^70^, ^71^, ^72^, ^73^, ^74^, ^75^, ^76^, ^81^, ^82^, ^83^, ^84^, ^85^, ^87^, ^88^, ^89^, ^91^, ^92^, ^93^, ^96^ Thirteen indices included family structure, often represented by variables related to female‐headed households or single head of households with children.^3^, ^15^, ^27^, ^33^, ^36^, ^38^, ^39^, ^40^, ^42^, ^78^, ^79^, ^80^, ^81^, ^86^, ^87^, ^88^, ^89^, ^91^, ^94^, ^95^, ^96^, ^98^ Eight indices included transportation, which was consistently represented by variables related to vehicle ownership.^27^, ^86^, ^87^, ^88^, ^89^, ^90^, ^91^, ^98^, ^99^

**TABLE 1:** Summary of nSES indices identified

Index/Author^a^ (variable selection method)	Domains							
	Income	Education	Employment	Housing	Transportation	Family structure	Demographic	Other
Area deprivation index^131^ (factor analysis)	(1) median family income; (2) income disparity; (3) % families below the poverty level; (4) % population <150% of the poverty threshold	(1) population aged >25 years with <9 years of education; (2) population aged >25 years with at least a high school diploma	(1) employed persons aged >16 years in white collar occupations; (2) civilian labor force population aged >16 years unemployed	(1) median home value; (2) median gross rent; (3) median monthly mortgage; (4) owner occupied housing units; (5) % households with more than one person per room	(1) % households without a motor vehicle	(1) % single‐parent households with children aged <18 years	—	—
Banegas index^29^ (a priori)	(1) household income; (2) poverty	(1) education	(1) occupation; (2) unemployment	(1) rent; (2) house values	—	—	—	—
Beyer index^97^ (a priori)	(1) median household income	(1) proportion without a high school diploma	(1) proportion unemployed	—	—	—	—	—
Concentrated affluence^43^ (a priori)	(1) % families with incomes above $75,000 (2000 Census period) or $50,000 (1990 Census period)	(1) % adults with college education	(1) % civilian labor force employed in professional/ managerial occupations	—	—	—	—	—
Concentrated disadvantage (2 variables)^30^ (PCA)^b^	(1) % below the poverty line	—	(1) % unemployed	—	—	—	—	—
Concentrated disadvantage (6 variables)^132^ (PCA)^c^	(1) % below the poverty line; (2) % receiving public assistance income	—	(1) % unemployed	—	—	(1) % female‐headed families	(1) % aged <18 years; (2) % Black	—
Coogan index^133^(PCA + factor analysis)	(1) median household income; (2) % households receiving interest, dividend or net rental income	(1) % adults aged ≥25 years that have completed college	(1) % employed persons aged ≥16 years that are in occupations classified as managerial, executive, or professional specialty	(1) median housing value	—	(1) % families with children not headed by single female	—	—
Diez‐Roux index^134^ (factor analysis)	(1) log of median household income; (2) % households receiving net rental, interest or dividend income	(1) % aged ≥25 years who completed high school and who completed college	(1) % employed aged ≥16 years in professional and managerial occupations	(1) log of median value of owner‐occupied housing units	—	—	—	—
Doubeni index^86^ (PCA)	(1) % below 1999 federal poverty levels; (2) % on public assistance; (3) % annual income of <$30,000	(1) % less than high school education	(1) % unemployed; (2) % men in managerial jobs; (3) % women in managerial jobs	—	(1) % no car	(1) % headed by a female with dependent children	(1) % non‐Hispanic Black	—
Dubowitz index^135^ (factor analysis)	(1) % below the poverty line; (2) % receiving public assistance; (3) median household income	(1) % aged ≥25 years with less than a high school education	(1) % male unemployment	—	—	(1) % households with children that are headed only by a female	—	—
ICE ‐ Income^136^, ^137^	(1) (n of persons in high‐income households)—(n of persons in low—income households)/total population with household income data	—	—	—	—	—	—	—
Johnson economic deprivation index^94^ (factor analysis)	(1) % below the poverty level; (2) % on public assistance	—	—	—	—	(1) % female head of house with children; (2) % married	—	—
Lian index^88^ (PCA)	(1) % receiving public assistance; (2) % low income; (3) % income no less than 400% of the US median household income; (4) median household income in 1999; (5) % below federal poverty line	(1) % less than a high school education; (2) % with a college degree	(1) % unemployed males aged ≥20 years; (2) % unemployed females aged ≥20 years; (3) % white collar; (4) % with low social class	(1) % households with ownership; (2) % vacant households; (3) % no less than 1 person per room; (4) median value of all owner‐occupied households; (5) % living in the same residence since 1995	(1) % households without a car	(1) % female‐headed households with dependent children	(1) % non‐Hispanic Black; (2) % Hispanic; (3) % residents aged ≥65 years	—
Material deprivation index^138^ (a priori)	(1) % living below the poverty level	—	(1) % aged ≥16 years unemployed	(1) % living in a crowded residence (more than 1 person per room)	(1) % households with no vehicle available	—	—	(1) % households with no telephone available
Messer index^22^ (PCA)	(1) % poverty; (2) % on public assistance; (3) households earning $30,000 per year estimating poverty	(1) % earning less than a high school education	(1) % males in management/ professional occupations; (2) % unemployed	(1) % crowded housing	—	(1) % female headed households with dependents	—	—
Mojica index^38^ (a priori)	(1) population receiving public assistance	(1) % without a high school diploma	(1) male population aged ≥16 who are unemployed	—	—	(1) households with children headed by females	—	—
Neighborhood deprivation index^139^ (PCA)	(1) % with income below the 1999 poverty status; (2) % income <$30 000 per year (3) % on public assistance income	(1) % did not graduate high school (age ≥25 years)	(1) % males and females who are unemployed;(2) % males in professional occupations	(1) % housing units with ≥1 occupant per room; (2) % occupied housing units with renter/owner costs >50% of income; (3) median household value	(1) % households with no car	(1) % female headed households with dependent children	—	—
Palmer index^39^ (factor analysis)	(1) median household income; (2) % households receiving interest, dividend, or net rental income	(1) % aged ≥25 years that have completed college	(1) % employed aged ≥16 years that are in occupations classified as managerial, executive, or professional specialty	(1) median housing value	—	(1) % families with children not headed by a single female	—	—
Reitzel index^98^ (a priori)	(1) % income below the poverty level in 1999	(1) % aged ≥25 years with less than high school degree/GED	(1) % aged ≥16 years unemployed	—	(1) % households with no vehicle available for use	(1) % single parent households	—	—
Social deprivation index^140^ (factor analysis)	(1) % in poverty	(1) % less than high school diploma	(1) % nonemployed	(1) % crowding; (2) % renter‐occupied housing	(1) % no car ownership	(1) % single parent households	—	—
Wheeler index^28^ (weighted quantile sum regression)	(1) median household income; (2) per capita income; (3) % households not on public assistance; (4) % families with children <18 years not in poverty; (5) Gini index of income equality	(1) % aged ≥25 years with a bachelor's degree	—	(1) % owner occupied housing; (2) % not vacant housing units; (3) median gross rent; (4) % households with mortgages	—	—	(1) % White	—
Yang index^141^ (PCA)	(1) % above 200% poverty line; (2) median household income	(1) Liu Education Index (% aged ≥25 years with college, high school and less than high school)	(1) % persons with a blue collar job; (2) % persons employed	(1) median rent; (2) median value of owner‐occupied housing units	—	—	—	—
Yost index^44^ (PCA^d^)	(1) median household income; (2) % below 200% of the poverty line	(1) Liu Education Index (% aged ≥25 years with college, high school and less than high school)	(1) proportion with a blue collar job; (2) % aged ≥16 years in the workforce without a job	(1) median rent; (2) median house value	—	—	—	—
Zhang index^90^ (PCA)	(1) % income below poverty; (2) % income <$22,500 (1990) or <$30,000 (2000); (3) % on public assistance	(1) % with less than a high school education	(1) % unemployed	—	(1) % households without a car	—	—	—

^a^ Indices that were created for use in one study only are named after the first author of the article in this table.

^b^ PCA, principal components analysis.

^c^ One paper utilized the six‐variable Concentrated Disadvantage Index but removed two of the variables (% households receiving public assistance income and % Black).^42^

^d^ The ICE—Income Index is described above. Additional ICE indices include ICE—Race/Ethnicity (*n* of “White non‐Hispanic” persons)‐(*n* of “black non‐Hispanic” persons)/*n* of persons with race/ethnicity data and ICE—Income + Race/Ethnicity (n of “White non‐Hispanic” high‐income persons)−(*n* of “black alone” low income persons)/*n* of persons with race/ethnicity and household income data.

### 3.2 nSES associations by cancer outcomes and cancer sites

**FIGURE 2:**
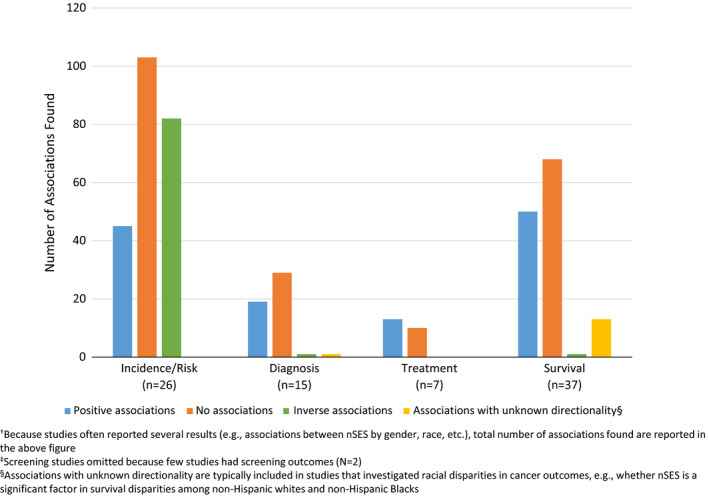
Number of associations^†^ found across studies by cancer control outcome^‡^

We first investigated whether statistically significant positive or inverse associations were reported for each index and cancer control outcome (incidence, diagnosis, treatment, survival/mortality). However, this analysis was limited because many indices were investigated in only one study. For indices used in more than one study and/or for more than one cancer outcome, there did not appear to be a consistent association between nSES and any of the cancer outcomes (Table S1). To further investigate whether potential patterns exist, associations were summed across all indices for each cancer outcome (Figure 2). No consistent association between nSES and overall cancer incidence/risk was observed. In contrast, studies predominately reported either a positive or no association for cancer diagnosis, treatment, and survival. These patterns generally remained: (1) Even when nSES indices with similar domains (*n* = 11 indices with income, education, employment, and housing domains) were summed and compared across cancer outcomes; (2) when restricting the analysis to only studies conducted in California (Table S3); or (3) when stratifying by only those studies that controlled for individual SES factors (e.g., education) or covariates such as smoking. Figures S1 and S2 show associations for incidence and survival by individual‐level adjustment (e.g., age), individual SES adjustment (e.g., race/ethnicity), and adjustments with covariates outside those included in SEER data (e.g., income, smoking).

A closer analysis of nSES associations with cancer incidence was conducted by cancer site. For breast cancer (*n* = 6 studies),^34^, ^39^, ^52^, ^53^, ^74^, ^92^ thyroid cancer (*n* = 1),^60^ and melanoma (*n* = 1),^31^ positive associations with nSES were commonly reported (breast: 11 positive associations/16 total^34^, ^52^, ^53^, ^74^; melanoma/thyroid: 2 positive associations/2 total^31^, ^60^) (Table S4). Inverse associations between nSES and incidence of cervical (*n* = 3 studies; 20 inverse associations/27 total)^51^, ^58^, ^74^ and lung (*n* = 7 studies; 70 inverse associations/123 total)^73^, ^74^, ^82^, ^85^, ^89^, ^91^, ^92^ cancer were more likely to be reported. No consistent associations emerged for prostate (*n* = 3 studies),^36^, ^74^, ^92^ colorectal (*n* = 6),^67^, ^74^, ^79^, ^86^, ^90^, ^92^ gastric (*n* = 2),^26^, ^76^ head and neck (*n* = 2),^51^, ^57^ and liver (*n* = 1)^47^ cancers (Table S4). No association between nSES indices and anal (*n* = 1 study; 9 no associations/10 total)^51^ and lymphoid (*n* = 2 studies; 62 no associations/86 total)^50^, ^75^ cancers were reported, but study number and sample sizes were low (Table S1).

Next, we explored associations of nSES with cancer diagnosis characteristics (e.g., stage, grade, aggressiveness, hormone receptor status). No consistent pattern emerged for any cancer, except colorectal cancer, where no association was most commonly reported (*n* = 1 study; 5 no associations/5 total).^38^ For cancer treatment, positive associations for lymphoid (*n* = 1 study; 4 positive associations/4 total)^61^ and lung (*n* = 1 study; 2 positive associations/2 total)^94^ cancers were observed. No clear patterns emerged in breast (*n* = 3 studies),^45^, ^54^, ^99^ ovarian (*n* = 1),^80^ and prostate (*n* = 1)^95^ cancers.

nSES was positively associated with survival in liver (n = 1 study; 4 positive associations/5 total),^66^ lymphoid (*n* = 2 studies; 2 positive associations/2 total),^62^, ^71^ head and neck (*n* = 2 studies; 5 positive associations/6 total), and ovarian (*n* = 1 study; 2 positive associations/2 total)^78^ cancers. No association between nSES and cancer survival/mortality was commonly reported in breast (*n* = 17 studies; 39 no associations/59 total)^10^, ^29^, ^33^, ^35^, ^37^, ^41^, ^46^, ^48^, ^54^, ^59^, ^64^, ^65^, ^66^, ^70^, ^77^, ^83^, ^87^ and kidney (*n* = 1 study; 4 no associations/5 total)^66^ cancers. No clear pattern of association between nSES and cancer survival/mortality was observed for prostate (*n* = 4 studies),^56^, ^66^, ^69^, ^83^ lung (*n* = 5),^66^, ^68^, ^83^, ^84^, ^94^ colorectal (*n* = 4),^66^, ^72^, ^83^, ^88^ and thyroid (*n* = 1)^63^ cancers (Table S4).

### 3.3 n **SES** associations by race/ethnicity

**FIGURE 3:**
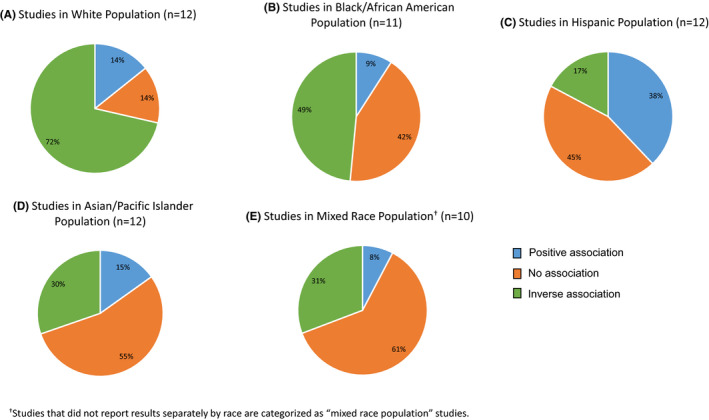
Associations between nSES and cancer risk/incidence by racial/ethnic group

A number of studies have observed that nSES and race/ethnicity are correlated and may independently and jointly impact cancer outcomes. Further, racial disparities often exist and continue to persist, even across low and high nSES.^24^, ^100^ This suggests that factors other than nSES play a role in contributing to minority health and health outcomes. Therefore, we examined nSES associations separately by racial/ethnic group to ensure associations were not being missed. Sixty percent (45/75 studies) reported associations by race/ethnicity.^46^, ^66^, ^85^ Among non‐Hispanic White cases (NHW; *n* = 12 studies), a trend emerged showing a clear inverse association between nSES and cancer incidence/risk (25 inverse associations, 5 no associations, 5 positive associations) (Figure 3). However, the protective benefits of nSES in relation to cancer incidence are diminished among other racial/ethnic groups.

**FIGURE 4:**
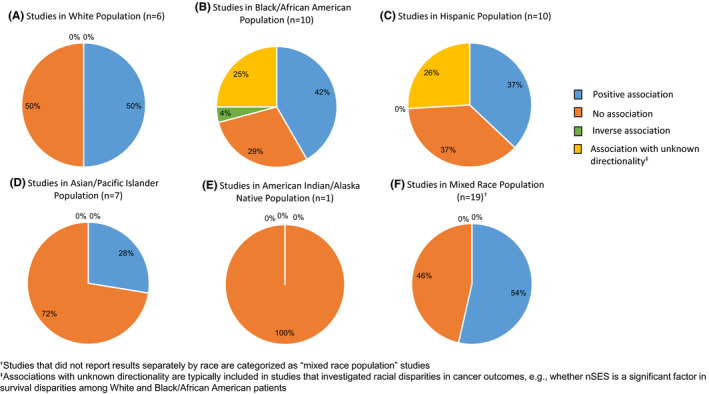
Associations between nSES and cancer survival by racial/ethnic group

A similar analysis was conducted for cancer survival, but a consistent pattern of association was not observed (Figure 4; Table S5). Across each race/ethnic group, approximately half of the findings reported no association and slightly fewer than half reported a positive association of higher nSES/improved survival.

We also conducted additional sub‐analyses to examine associations between nSES and cancer site‐specific outcomes by race/ethnicity (Table S6). The number of studies was generally too small to analyze for most cancers, but the following trends were observed. Among Hispanic cases, nSES was positively associated with breast cancer incidence (*n* = 4 studies; 5 positive associations/5 total) but inversely associated with cervical cancer incidence (*n* = 3 studies; 5 inverse associations/5 total). For lung cancer incidence (*n* = 7 studies), inverse associations were consistently reported for NHW cases (*n* = 12 inverse associations/14 total), Black cases (*n* = 10 inverse associations/14 total), and Asian cases (n = 7 inverse associations/12 total). For colorectal cancer incidence (*n* = 6 studies), nSES was inversely associated in NHW cases (4 inverse associations/4 total) but positively associated in Hispanic cases (2 positive associations/2 total). For head and neck cancer incidence, inverse associations were reported in NHW cases (*n* = 1 study; 5 inverse associations/6 total); whereas no associations were found among Asian cases (*n* = 2 studies; 11 no associations/18 total).

## 4 DISCUSSION

In this review, we found the relationship between nSES indices and cancer outcomes varied by cancer site and race/ethnicity. In general, nSES was inversely associated with cancer incidence/risk among NHW cases, but this association was less consistent in other race/ethnic groups. nSES was positively associated with cancer survival only for select cancers. Among more common cancers (prostate, colorectal, lung), no clear patterns emerged. These findings highlight the complex association between nSES and cancer control outcomes.

### 4.1 n **SES indices**

Twenty‐four nSES indices were identified, and the construction of nSES indices varied across studies. The majority included domains related to income, education, employment, and housing and utilized similarly representative variables. Notably, the relationship between nSES and cancer outcomes did not necessarily change by nSES variable selection. When we compared cancer incidence and survival findings of nSES indices that only included these four domains to those that also included transportation, family structure, additional demographics, etc., we did not see different associations across indices. This suggests nSES measures are similar in what they capture, and thus it might not matter which nSES measure is used in association studies. However, the majority of studies did not compare across nSES indices within the same study and selection of standard nSES measures may be preferred moving forward to allow for consistency and comparability across studies. Furthermore, the majority of studies did not consider the geographic distribution of the disease to account for the possibility that nearby neighborhoods are more likely to be similar to one another. Recent geospatial cluster analyses show some nSES measures may be more effective than others at explaining the geospatial distribution of cancer within a particular state,^10^ and that differences in nSES by neighborhood or geographic location, including living in an urban versus rural area,^101^ could affect cancer mortality.^102^ This suggests additional studies that consider spatial associations, urbanicity, and evaluate more than one existing nSES index are warranted before particular nSES measures can be recommended as a standard (Table 3).

### 4.2 Cancer incidence

In the aggregate, there were no consistent relationships between nSES indices and overall cancer incidence. Findings for cancer incidence remained unchanged even when restricting analyses to studies with additional adjustments for individual‐level factors, including education and income, which are known to be associated with both nSES and cancer outcomes (Table S1).^36^, ^39^, ^52^, ^53^, ^79^, ^92^ Because combining all cancer types could be masking potential associations, site‐specific analyses subsequently revealed positive associations of nSES with breast, thyroid, and melanoma cancer incidence. Analyses by race/ethnicity also demonstrated an inverse association of nSES with cancer incidence for NHW cases, reflecting a protective role in this population. This is consistent with prior research demonstrating that neighborhood disadvantage can adversely impact cancer risk through various pathways, including limited access to high‐quality diet, fewer opportunities for outdoor recreation and physical activity, and environmental exposures.^103^, ^104^

Although the sample size and the number of studies were limited for race/ethnicity‐specific analyses, our findings revealed positive associations of nSES with breast and colorectal cancer incidence among Hispanic cases. Living in higher SES neighborhoods may reflect greater acculturation and adoption of U.S. lifestyle behaviors. Acculturation has been correlated with key breast cancer risk factors including later age at first birth, having fewer children, shorter duration of breastfeeding, and increased alcohol consumption.^105^, ^106^ In contrast, an inverse association emerged for nSES and cervical cancer incidence among Hispanic women. In this context, greater acculturation may be beneficial as it has been associated with greater cervical cancer screening uptake.^107^

An inverse association was also observed between nSES and lung cancer incidence among NHW, Black, and Asian cases. Given the impact of environmental factors (e.g., smoking,^89^ air pollution exposure^108^) on this cancer, it is likely that low nSES correlates with greater exposure to these risk factors, but additional studies are warranted to tease these associations apart.

Overall, the stratified findings indicate the benefits of higher nSES for cancer incidence can be attenuated for racial and ethnic minoritized groups, particularly by cancer site. There may be several explanations for this finding. First, it is well‐documented that systemic and structural racism and discrimination against members of racial/ethnic minoritized groups occurs in healthcare,^109^, ^110^, ^111^, ^112^ regardless of the patient’s socioeconomic status or neighborhood residence. These biases, in turn, impact patient‐provider interactions, decision making and access to treatments, and healthcare utilization,^109^, ^110^, ^111^, ^112^ all of which have subsequent downstream effects on health outcomes. Further, discrimination against race/ethnic groups can also influence place of residence. Choice of residence is not always voluntary and may be driven by financial resources^113^, ^114^ or policies. Over several decades, policies on various scales intentionally created racial segregation through housing development and financial programs and shaped the demographics of the neighborhoods (e.g., red‐lining).^115^ These red‐lining policies are examples of structural racism that could be contributing to the attenuation of cancer incidence outcomes in race/ethnic groups from both high and low SES areas.^116^, ^117^

Second, nativity may play an important role as well. Ethnically dense neighborhoods (e.g., ethnic enclaves), some of which are low‐SES neighborhoods with large immigrant populations, report better health behaviors associated with cancer, including diet.^118^ As a result, the maintenance of healthy behaviors may help offset some of the adverse effects of low nSES, particularly among immigrant and poorer populations. Third, the frequency of nSES variables or domains used to represent common components in nSES indices may differ across race/ethnic groups, subgroups, and geographic location in a way that can impact disease associations. For instance, a higher proportion of Black and Hispanic patients compared to NHW patients often live in neighborhoods with lower income and higher poverty; thus, the impact of nSES indices may be attenuated when comparing within versus across race/ethnic groups. As such, it should be noted that studies of minority populations often have smaller sample sizes than studies of NHW populations, which could adversely influence the ability to detect statistically significant associations. Finally, previous studies have shown that different nSES domains may have differential effects by race/ethnicity. For instance, an empiric study of independent measures of nSES found that economic (e.g., income, poverty) and transportation measures were associated with advanced prostate cancer in White men, whereas housing measures were associated with advanced disease in Black men.^119^ These findings suggest indices that equally‐weight nSES domains may be masking important neighborhood effects in racial/ethnic minoritized populations;^120^ however, additional studies are needed. In particular, standardized methodologic assessments of existing indices are warranted, particularly before creating potentially new indices. More specifically, pooled analyses from multiple states (to increase sample size) that evaluate more than one nSES index within a single study AND that evaluate individual domains within that index, overall and by race/ethnicity, would be suggested to determine what indices or domains may be impacting observed associations. These analyses should further include adjustments for nativity when these data are available to help elucidate true nSES effects, which will aid in the standardized selection of nSES indices, as well as provide insights into drivers of disparities in cancer incidence (Table 2).

**TABLE 2:** Summary of geographic locations and cancer control outcomes studied within nSES indices

Index/author	States/Regions	Total studies (*N*)^a^	Number of studies by cancer control outcome				
			Risk/incidence (*N*)	Screening (*N*)	Diagnosis (*N*)	Treatment (*N*)	Survival/mortality (*N*)
Yost index^44^	CA (*n* = 37); SEER‐18 participating regions (*n* = 1); National (*n* = 1)	40	16	—	6	3	22
Concentrated disadvantage (6 variables)^132^	IL (*n* = 4); LA (*n* = 2)	6	1	—	3	1	2
Messer index^22^	AR, KY, MS, SC, TN, VA, WV (*n* = 1 each); CA, MI, NJ (*n* = 2 each); FL, GA, LA, NC, PA (*n* = 3 each)	4	1	—	2	—	2
Yang index^141^	CA	4	2	—	—	—	2
Concentrated affluence^43^	IL	3	—	—	1	1	1
Diez‐Roux index^134^	WA	2	1	—	—	—	1
Lian index^88^	CA, FL, GA, LA, MI, MO, NC, NJ, PA (*n* = 1 each)	2	—	—	—	—	2
Area deprivation index^131^	OH	1	1	—	—	—	—
Banegas index^29^	CA	1	—	—	1	—	1
Beyer index^97^	National (100 metropolitan areas)	1	—	—	—	—	1
Concentrated disadvantage (2 variables)^30^	IL	1	—	—	1	—	—
Coogan index^133^	Southeastern US (AL, AR, FL, GA, KY, LA, MS, NC, SC, TN, VA, WV)	1	—	—	—	—	1
Doubeni index^86^	6 US states (CA, FL, LA, NJ, NC, PA) or 2 metropolitan areas (Atlanta, Georgia; Detroit, Michigan)	1	1	—	—	—	—
Dubowitz index^135^	PA	1	—	—	—	1	—
ICE ‐ income^136^, ^137^	NJ	1	—	—	—	—	1
Johnson economic deprivation index^94^	GA	1	—	—	—	1	1
Material deprivation index^138^	MI	1	—	—	—	1	—
Mojica index^38^	CA	1	—	—	1	—	—
Neighborhood deprivation index^139^	AL, AR, FL, GA, KY, LA, MS, NC, SC, TN, VA, WV	1	1	—	—	—	—
Palmer index^39^	CA, GA, IL, MA, NJ, NY, VA, Washington DC	1	1	—	1	—	—
Reitzel index^98^	LA; TX	1	—	—	—	—	1
Social deprivation index^140^	VA	1	—	1	—	—	—
Wheeler index^28^	MN; WI	1	—	1	—	—	—
Zhang index^90^	CA, FL, GA, LA, MI, NJ, NC, PA	1	1	—	—	—	—

^a^ Because several studies utilized the same nSES index for multiple cancer control outcomes, the number of studies listed across the cancer control outcomes may not add up to the total studies.

### 4.3 Cancer survival

Overall, no clear pattern of associations of nSES with diagnosis or treatment emerged, perhaps due to the heterogeneity of disease staging approaches across cancer sites. nSES was positively associated with cancer survival for selected cancers (liver, lymphoid, head, and neck, ovarian). Previous literature has reported higher individual‐level SES (e.g., education, income, insurance coverage) often correlates with higher nSES.^13^ As a result, residents of higher SES neighborhoods may have greater access to health care resources and healthier foods, which could lead to positive associations with cancer outcomes because individuals from high SES backgrounds and environments are more likely to receive timely cancer treatment and follow‐up care.^121^, ^122^

On the other hand, no association between nSES and cancer survival was observed for breast cancer, and a clear association could not be established for prostate, lung, and colorectal cancers. This could be due to the small number of studies conducted within these sites, or it could be reflective of studies being conducted across multiple‐year ranges (e.g., breast cancer survival studies ranged from 1988 to 2014) or in a single state (e.g., 14/17 breast cancer survival studies were conducted in California). Notably, there was no clear association between nSES and different cancer outcomes within one cancer. For instance, nSES was positively associated with breast cancer incidence, but generally not breast cancer survival. This suggests that nSES exerts differential effects, not just by cancer site, but within a cancer site, across the disease continuum. Thus, future studies are needed to investigate the longitudinal trajectory of nSES on outcomes from cancer incidence to survival.

### 4.4 Limitations and additional recommendations for future studies

Several limitations should be noted (Table 3). First, given the heterogeneity in defining nSES and cancer outcomes themselves, potential publications may have been missed, despite our comprehensive search strategy. Second, publication bias can result from the tendency of authors to only publish studies with significant results and is a cited limitation of most systematic reviews. Although the inclusion of grey literature is sometimes suggested to address this bias and aid in validating the results of a literature search of published research, there are also disadvantages in that these sources are often not peer‐reviewed, may not report relevant information, and have the potential for introducing additional bias. To address the question of when grey literature should be included, Benzies et al provided a checklist to help guide authors’ decisions.^123^ Using this checklist, we determined that the availability of studies on the impact of nSES on cancer outcomes is of high volume and similar quality, with studies often utilizing similar data resources (e.g., cancer registry and U.S. Census variables for the general of nSES indices) for study measures. Through these steps, we concluded that the focus on published, peer‐reviewed data was justified. Further, the results of this systematic review showing variation in associations overall (including many reports of null associations) and by factors known to affect associations with nSES and cancer (e.g., race/ethnicity in SEER registry studies), suggest publication bias may be minimized in this body of literature. Notably, in studies where more detailed risk factors for cancer were available (e.g., studies that utilized cohort data with detailed smoking data), patterns and associations with cancer overall and by race/ethnicity continued to remain. Third, the majority of nSES indices were constructed at the census tract level, which is considered to be an adequate geographic level with which to look for associations with disease;^25^ but research on the incorporation of daily activity spaces, of how people in a particular neighborhood move and interact with their local geographies, is also needed.^124^, ^125^ This is because while the use of administrative boundaries, like census tracts, allows for consistency in reporting across US studies, they may not adequately represent where people spend their time^7^ or what neighborhood environments they are exposed to. Fourth, the majority of studies reviewed were conducted in California, a national leader in the evaluation of nSES and cancer outcomes which can serve as a model for other states.^126^ However, in order to move towards standardized nSES index measures, studies across more US states are needed, given that the variation in nSES indices and their associated variables likely differ by geography.^102^ Fifth, the majority of reviewed studies utilized State Cancer Registries. Increasing the number of state‐specific analyses within and across cancer sites could help clarify the role of nSES in cancer outcomes. Given cancer registry and U.S. Census data are readily available, efforts to support investigations into the role of nSES in cancer outcomes within and across states are warranted and in line with initiatives to incorporate standardized disparity measures in future cancer studies. More accessible mechanisms for investigators to link their own custom nSES indices to multistate cancer registry datasets like SEER or North American Association of Central Cancer Registries (NAACCR) data would help advance investigations into the role of nSES in cancer outcomes.

**TABLE 3:** Recommendations for future association studies in cancer to aid in variable selection and studies of health disparities

Evaluate more than one nSES index in association studies Consider evaluating associations in both statistical and geospatial studies Include summary statistics on each domain within selected indices by race/ethnicity to evaluate if domains within indices might differentially impact or drive associations by race/ethnicity Expand nSES and cancer‐site specific studies to include additional States and pooled analyses; Data is limited for a number of cancer sites and states beyond California Conduct additional studies focused on nSES, cancer screening, and diagnosis Evaluate the impact of nSES within a single cancer site across the continuum (diagnosis, stage, treatment [type and time to treatment], survival) Implement multilevel studies across the cancer continuum that evaluate nSES in the context of: Clinical data from electronic medical records or health studies Nativity/ethnic enclaves/segregation Urban/rural designations Race/ethnic groups and subgroups Methods recommendations: Explore alternative geographic boundaries using daily activity data Incorporate information about residential history to allow for investigations of the impact of nSES over the lifespan Report nSES associations by race/ethnic group and subgroup

To further elucidate potential etiologic effects, additional studies are needed to investigate nSES associations with cancer screening and diagnosis. This is crucial for determining where along the continuum nSES may exert effects. For example, it is possible that nSES may have more of an impact on cancer development and stage at diagnosis, due to differential exposures and access to care, but it may have less of an impact on survival, particularly among patients diagnosed with metastatic disease.^127^ More generally, studies that investigate the role of nSES across the continuum for specific cancers and that can incorporate relevant individual‐level behaviors, race/ethnicity, and clinical factors are needed. This would involve the extension of current nSES research beyond just the use of registry data to incorporate nSES data with electronic medical records, and other available cohort and case‐control studies.

To expand on etiologic work, studies investigating the effect of residential history (e.g., change in nSES over time) are beginning to emerge. The role of residential history may be particularly relevant to the Hispanic paradox and socio‐spatial mobility (i.e., movement between neighborhoods with foreign‐born and U.S.‐born residents or various nSES). Prior studies have just utilized nSES at the time of diagnosis, without consideration of lifetime nSES exposures, which may change over time.^128^, ^129^ More recent studies have shown that not only does residential history vary by race/ethnicity, but also that the pattern of nSES moves (e.g., moving from high to low nSES areas) may influence cancer survival outcomes.^128^, ^129^, ^130^ Many of these studies utilize poverty as the main measure of nSES change, but the differential pattern of findings by race/ethnicity in the present study suggests that other measures, including change in segregation or movement in/out of ethnic enclaves over time, should also be explored. This suggestion is consistent with our recommendation to evaluate multiple nSES indices in state/national studies going forward, given the differences noted by race/ethnicity and geographic levels, in order to aid in more standardized variable selection. To further support etiologic investigations, changes in nSES and length of time spent in an unfavorable nSES environment should continue to be explored, given that chronic exposure to unfavorable circumstances over a long time period (10 years or more) may be needed for chronic disease development, like cancer.^128^, ^129^

## 5 CONCLUSION

This comprehensive review yielded a searchable, publically available database that can be used by researchers designing future studies centered on nSES and cancer. These findings highlight methodologic and conceptual approaches surrounding the measurement of nSES that can inform nSES variable selection in future studies and help clarify its role in contributing to cancer disparities. The use of different nSES indices (and different variables to form these indices) across geographic locations, study cohorts, cancer sites, and outcomes complicates the field’s ability to draw meaningful conclusions about nSES as a standard risk factor for cancer outcomes. Given the lack of consensus regarding appropriate measures of disparity, including optimal variables to include in index construction, this study has recommended approaches for evaluating different nSES measures within and across cancer sites, overall and by race/ethnic group, utilizing additional state/national cancer registries to help standardize variable selection in future studies. Utilization of a standard nSES index would aid in etiologic and intervention research related to cancer health disparities. Furthermore, this study highlights the need for additional studies in population and clinical datasets that couple nSES measures with more detailed clinical and behavioral variables to enable the evaluation of the true impact of nSES on cancer health disparities.

## CONFLICT OF INTEREST

The authors declare no conflicts of interest.

## DATA SHARING AND ACCESSIBILITY

The data underlying this article are available in Supporting Files.

## Supporting information


Figure S1.

Figure S2.

Table S1.

Table S2.

Table S3.

Table S4.

Table S5.

Table S6

## Data Availability

The data underlying this article are available in Supporting Files (https://github.com/ksorice/nSES‐Systematic‐Review).
