# Supplementary material for: Systematic review of neighborhood socioeconomic indices studied across the cancer control continuum
Source: Cancer Med. 2022 Feb 14;11(10):2125–44. doi: 10.1002/cam4.4601 (PMC9119356; doi:10.1002/cam4.4601)
Supplement: Supplementary file 1 — Figure S1. [file CAM4-11-2125-s007.pdf]

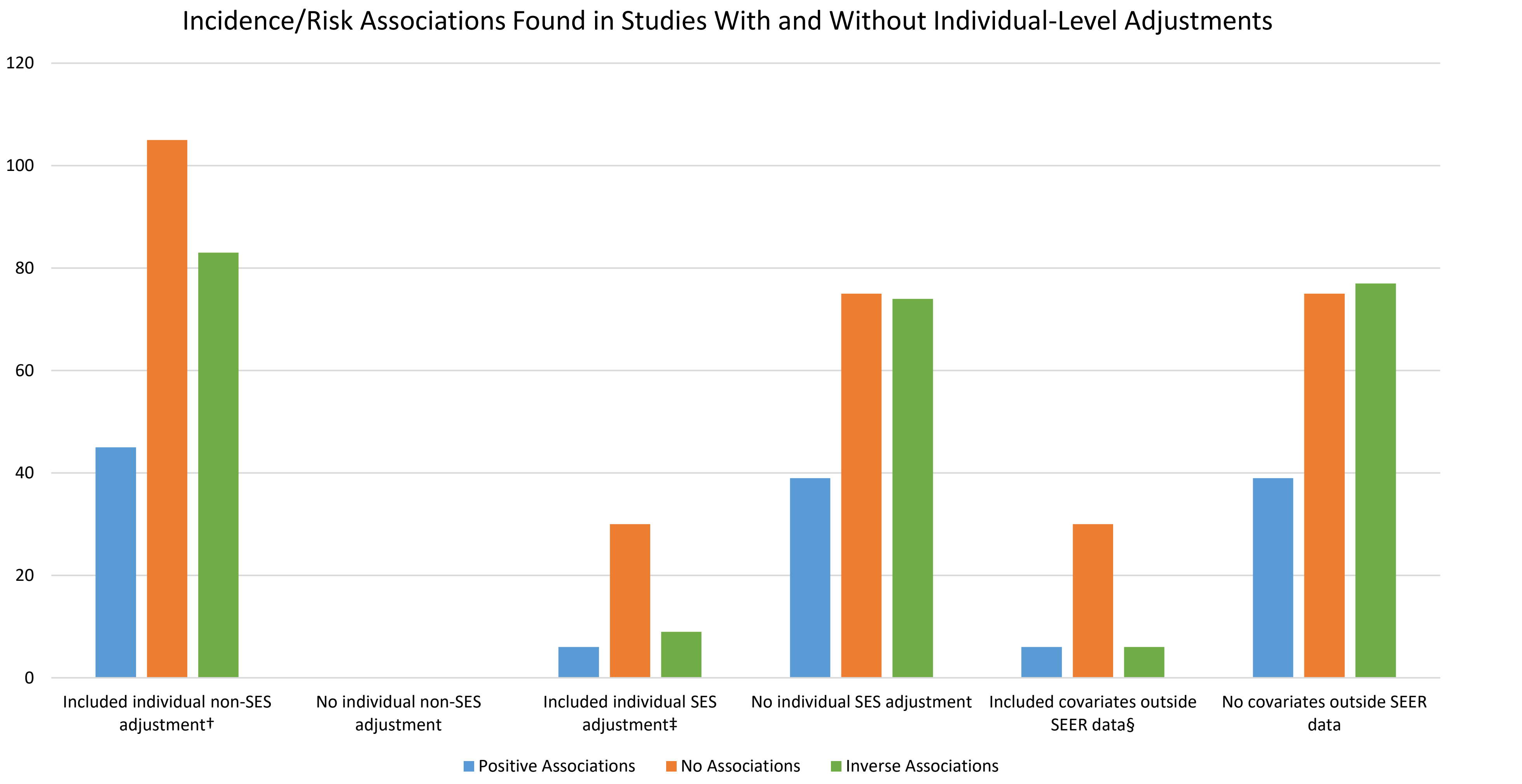

†Individual non-SES adjustments include non-SES variables that are typically included in SEER registries (e.g., age, diagnosis year). All incidence studies included at least one individual level non-SES adjustment.

‡Individual SES adjustments include race/ethnicity, education, etc.

§Covariates outside variables included in SEER data include both SES variables (e.g., income) and non-SES variables (e.g., smoking history).
